# A PROTAC peptide induces durable β-catenin degradation and suppresses Wnt-dependent intestinal cancer

**DOI:** 10.1038/s41421-020-0171-1

**Published:** 2020-06-09

**Authors:** Hongwei Liao, Xiang Li, Lianzheng Zhao, Yalong Wang, Xiaodan Wang, Ye Wu, Xin Zhou, Wei Fu, Lei Liu, Hong-Gang Hu, Ye-Guang Chen

**Affiliations:** 1 The State Key Laboratory of Membrane Biology, Tsinghua-Peking Center for Life Sciences, School of Life Sciences, Tsinghua University, 100084 Beijing, China; 2 School of Pharmacy, Second Military Medical University, 200433 Shanghai, China; 3 Department of General Surgery, Peking University Third Hospital, Beijing, China; 4 Tsinghua-Peking Center for Life Sciences, Department of Chemistry, Tsinghua University, 100084 Beijing, China; 5 Institute of Translational Medicine, Shanghai University, 200444 Shanghai, China

**Keywords:** Drug development, Targeted therapies

## Abstract

Aberrant activation of Wnt/β-catenin signaling has been associated with the onset and progression of many types of tumors and thus β-catenin represents one attractive intracellular target for cancer therapy. Based on the Axin-derived peptide that binds to β-catenin, two stapled peptides SAHPA1 and xStAx were reported to enhance or impair Wnt/β-catenin signaling, respectively. In this study, we designed PROTACs (proteolysis targeting chimeras) by coupling SAHPA1 or xStAx with the VHL ligand to achieve efficient β-catenin degradation. The obtained xStAx-VHLL sustained β-catenin degradation and manifested strong inhibition of Wnt signaling in cancer cells and in *APC*^*−/−*^ organoids. Furthermore, xStAx-VHLL could effectively restrain tumor formation in BALB/C nude mice, and diminish the existing tumors in *APC*^*min/+*^ mice. More importantly, xStAx-VHLL could potently inhibit the survival of colorectal cancer patient-derived organoids. These findings suggest that xStAx-VHLL exhibits the ability of cancer prevention and cure, highlighting the potential of β-catenin degrader PROTACs as a new class of promising anticancer agent.

## Introduction

The Wnt/β-catenin signaling pathway plays a pivotal role in cell proliferation, differentiation, and survival^1,2^. The components of canonical Wnt signaling consist of extracellular Wnt ligands, transmembrane receptors Frizzled/LRP5/6 (low-density lipoprotein receptor-related protein), the intracellular destruction complex (including APC, Axin, CK1, GSK3β, and β-TrCP) and β-catenin^3^. As the canonical Wnt signal transducer, the stability of β-catenin is tightly controlled by the interplay between Wnt ligands, receptors and the destruction complex^2^. Once mutations occur to Wnt signaling components, excessive β-catenin accumulates in the cytoplasm and translocates into the nucleus to promote cell proliferation^4^. Aberrant activation of Wnt signaling is highly implicated in various types of cancer, including colorectal cancer, in human, for example, hotspot mutations of APC, Axin, and β-catenin have been found to drive tumorigenesis^2^. Hence, inhibition of the Wnt/β-catenin pathway has already become an important approach for cancer therapy, and β-catenin is the most attractive intracellular target^5^.

Many small-molecule inhibitors of Wnt/β-catenin signaling have been identified^6^. However, none of them has been approved for the clinical use. As β-catenin is the central player of the canonical Wnt signaling and is frequently mutated in cancers, it is the most attractive target for cancer therapy. For instance, PRI-724, which can disrupt the interaction between β-catenin and its co-activators, has been tested for clinical use^7^. One of the caveats of this type of inhibitors could be insufficient to disturb all the protein–protein interactions due to the large binding interface and complicated binding partners in heterogeneous tumor tissue. Therefore, it is ideal to design inhibitors that can induce β-catenin degradation^4,8^. The small-molecule MSAB is a such example^9^, although its specific binding site and the working mechanism are still unclear.

Peptides are superior as therapeutics to small molecules in interacting with β-catenin but still suffer from the poor membrane permeability and protease stability^10^. Peptide stapling chemistry developed by Verdine and coworkers, takes advantage of ruthenium-catalyzed metathesis to crosslink the side chains of paired anchoring residues at suitable positions, and has been successfully applied to design various peptide inhibitors with improved proteolytic stability, membrane permeability, and thus biological activity^11,12^. Modeled after the same Axin-derived peptide motif, we and Verdine lab independently designed and prepared two different stapled helical peptides, termed as SAHPA1 and xStAx, respectively^13,14^. Both SAHPA1 and xStAx are capable of penetrating the cell membrane and binding with β-catenin. Interestingly, SAHPA1 was shown to activate Wnt/β-catenin signaling, while xStAx inhibited it^13,14^. The opposite effect of these peptides could be due to their different working mechanisms: xStAx blocks the β-catenin-TCF interaction in the nucleus, while SAHPA1 enhances the dissociation of β-catenin from Axin in the presence of Wnt. Nevertheless, it still remains challenging to drive xStAx efficiently inhibit Wnt-dependent tumor growth in cell culture, and importantly, in vivo, due in part to the rapid accumulation of β-catenin in cancer. To overcome these limitations of β-catenin inhibitors, new strategies are urgently in need to achieve the long-lasting degradation of β-catenin and thus tumor regression.

In recent years, proteolysis-targeting chimaeras (PROTACs) has emerged as a useful technology to efficiently degrade protein of interest in vivo^15–17^. PROTACs consist of two ligands connected by a linker, with one ligand specifically binding to the protein of interest and the other ligand recruiting an E3 ligase. When PROTACs interact with the target protein and an E3 ligase concurrently, the target protein will be poly-ubiquitinated and then undergo proteasomal degradation. Compared with traditional inhibitors that need a 1:1 (inhibitor:target) activity ratio, PROTACs can continuously function and show sub-stoichiometric property^18,19^. For instance, a stabilized peptide-based PROTAC has been successfully developed to target estrogen receptor α for the efficient degradation^20^.

In this study, to achieve efficient elimination of β-catenin, we designed novel PROTAC β-catenin degraders by coupling SAHPA1 or xStAx with VHL (von Hippel-Lindau protein) ligand via an Ahx chemical linker, termed as SAHPA1-VHLL and xStAx-VHLL, respectively. We found that xStAx-VHLL manifested strong inhibition of Wnt signaling and sustained degradation of β-catenin in cancer cells and the intestinal organoids derived from wild-type and *APC*^–/–^ mice. xStAx-VHLL also restrained tumor formation in xenograft mouse models and reduced intestinal tumors in *APC*^*min/+*^ mice. Besides, xStAx-VHLL potently inhibited the survival of the CRC patient-derived organoids. This study is the first attempt to block Wnt signaling via PROTAC-mediated β-catenin degradation, highlighting the potential of PROTAC peptides as a new class of promising agents against the diseases caused by overactivation of Wnt/β-catenin signaling.

## Results

### Design of PROTAC β-catenin degraders

Based on the observation that the β-catenin binding domain of Axin (amino acid 469–482) forms a stable continuous α-helix and fits into a shallow groove of β-catenin^21^, we and Verdine group designed stapled helical peptides that specifically targets β-catenin to modulate Wnt signaling^13,14^. Although two of the stapled peptides (SAHPA1 and xStAx) shared high similarity in sequences, SAHPA1 acted as an agonist for Wnt signaling while xStAx functioned to inhibit the pathway.

**Fig. 1:**
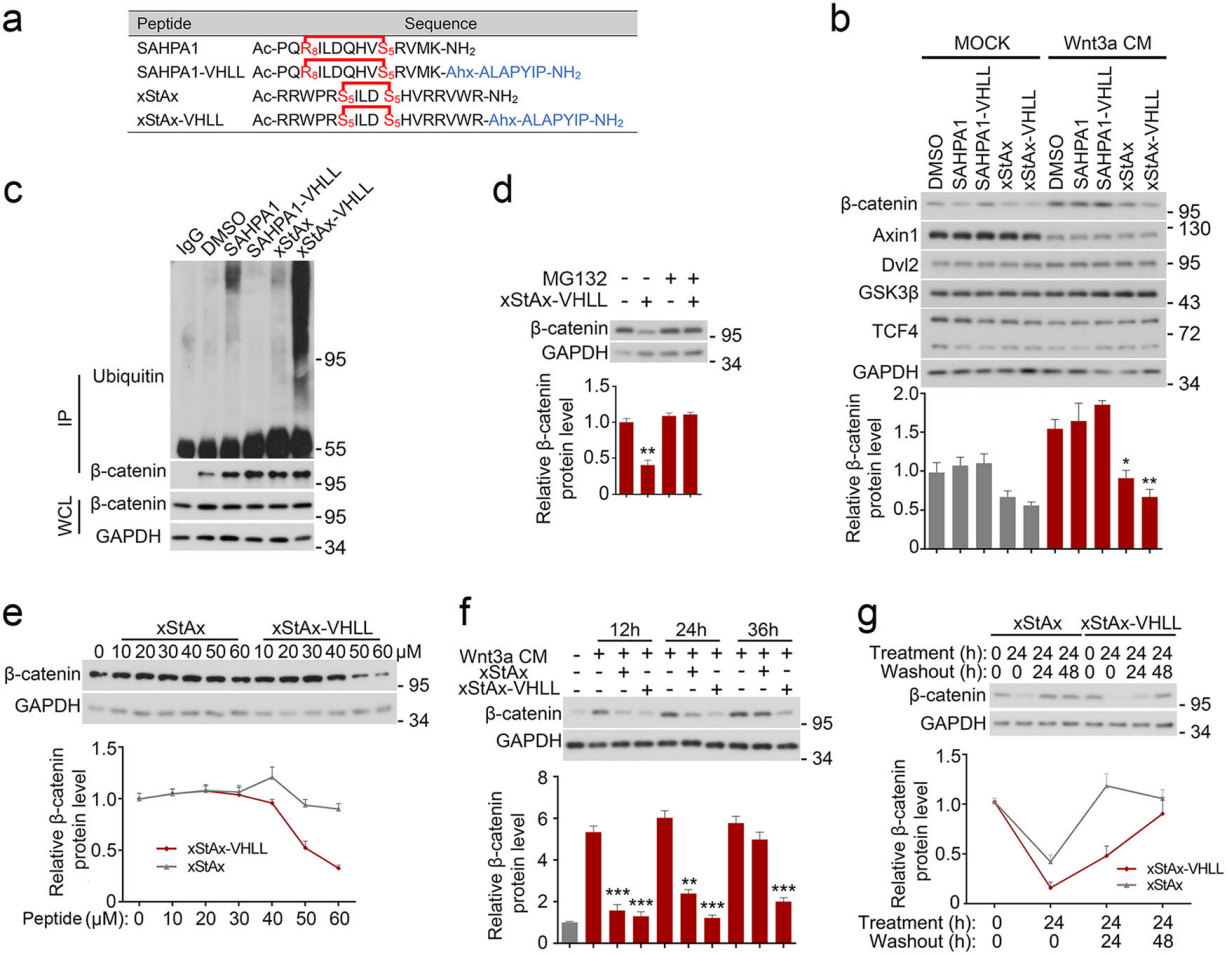
xStAx-VHLL promotes the proteasomal degradation of β-catenin. **a** The amino acid sequence of the designed peptides. **b** HEK293T cells were treated with Wnt3a conditioned medium (CM), the vehicle (DMSO) or various peptides (70 μM) as indicated for 24 h and then harvested for immunoblotting. Relative β-catenin protein level was quantified as shown below. **c** HEK293T were cells treated with peptides (50 μM) for 24 h and MG132 (10 μM) for 12 h before harvested for immunoprecipitation (IP) and immunoblot. Protein expression was confirmed with whole cell lysates (WCL). **d** HEK293T cells were treated with peptides (70 μM) and the proteasome inhibitor MG132 (10 μM) for 16 h and then harvested for immunoblotting. **e** HCT116 cells were treated with the indicated concentration of the peptides for 24 h to detect the dose dependent degradation of β-catenin. **f** HEK293T cells were treated with peptides (70 μM) and Wnt3a CM for indicated time and then harvested for immunoblotting. **g** HCT116 cells were treated with peptides (50 μM) for 24 h and then replaced with fresh medium for 24 or 48 h to wash out the peptides before harvested for immunoblotting. The relative band intensity was quantified with ImageJ and normalized to GAPDH. Data from three independent experiments are displayed as the mean ± SD by one-way ANOVA. *P < 0.05, **P < 0.01.

As both SAHPA1 and xStAx can specifically bind to β-catenin, we made use of these stapled peptides as the tethering site for the design of bifunctional PROTAC β-catenin degraders. The VHL recognition peptide sequence, ALAPYIP that has already been widely used in the design of peptide-based PROTAC degraders^16^, is employed as a ligand for the VHL E3 ligase recruitment. SAHPA1 or xStAx is conjugated to the VHL ligand via amide bonds with a simple 6-Aminocaproic acid as the linker, generating the two PROTAC peptides: SAHPA1-VHLL and xStAx-VHLL (Fig. 1a and Supplementary Fig. S1a). After HPLC purification, the synthesized PROTACs were readily obtained with over 95% purities, as verified by ESI-MS (Supplementary Fig. S1b).

As determined by isothermal titration calorimetry (ITC) and pulldown assay, stapled peptides and the corresponding PROTACs displayed comparative binding potency with β-catenin (Supplementary Fig. S2). Furthermore, both xStAx and xStAx-VHLL had similar membrane permeability and could efficiently enter into a cell (Supplementary Fig. S3a), but xStAx-VHLL was retained in the cell much longer (Supplementary Fig. S3b).

### xStAx-VHLL induces dose-dependent and durable β-catenin degradation

Then we examined the activity of these PROTAC peptides to induce degradation of the endogenous β-catenin protein. After treated with 70 μM peptides for 24 h, HEK293T cells were harvested for immunoblotting. As shown in Fig. 1b, SAHPA1 slightly enhanced the β-catenin level while xStAx decreased it. These results were in agreement with the previous reports^13,14^. Similarly, xStAx-VHLL also reduced the β-catenin level. However, xStAx-VHLL significantly downregulated β-catenin protein level, which was obviously lower than xStAx, when the cells were treated with Wnt3a (Fig. 1b). Of note, these peptides had no effect on other Wnt signaling components as examined. Similar data were obtained with colorectal cancer (CRC) cells, including HCT116, SW480, and LoVo cells (Supplementary Fig. S4). HCT116 cells harbor β-catenin mutation, while both LoVo and SW480 cells have *APC* mutation^22^. As expected, xStAx-VHLL-induced reduction of β-catenin was mediated by the ubiquitination-proteasome system as xStAx-VHLL promoted β-catenin ubiquitination and the proteasome inhibitor MG132 blocked β-catenin degradation (Fig. 1c, d). The VHL ligase activity was required for xStAx-VHLL to mediate β-catenin degradation as the VHL ligase inhibitor VH-298 blocked it in HEK293T and LoVo cells (Supplementary Fig. S5a, b). We also found that StAx-VHL could induce β-catenin degradation in a dose-dependent manner in CRC cells (Fig. 1e, Supplementary Fig. S5c, d). Both xStAx and xStAx-VHLL started to induce β-catenin degradation as soon as 12 h post treatment. However, only xStAx-VHLL maintained β-catenin at a low level up to 36 h (Fig. 1f). The wash-out experiment indicated that β-catenin was remained low after 24 h of xStAx-VHLL removal while β-catenin level was restored after 24 h of xStAx removal (Fig. 1g). Therefore, these results suggested that xStAx-VHLL was capable of achieving strong and sustained β-catenin degradation.

### xStAx-VHLL inhibits Wnt/β-catenin signaling

**Fig. 2:**
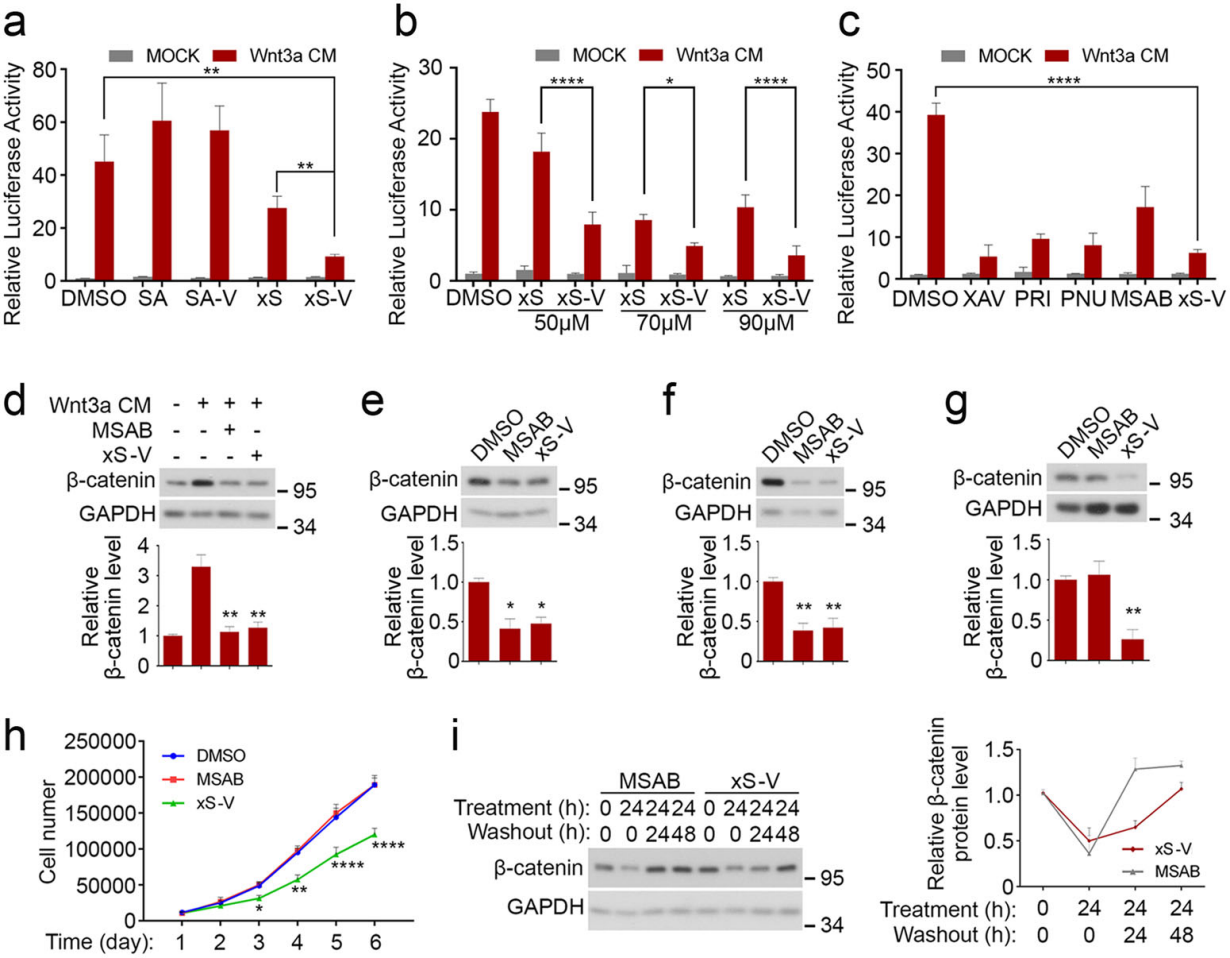
xStAx-VHLL inhibits Wnt/β-catenin signaling. **a** HEK293T cells transfected with Topflash luciferase were treated with Wnt3a CM and peptides (70 μM each) for 24 h and then harvested for luciferase measurement. SA SAHPA1, SA-V SAHPA1-VHLL, xS xStAx, xS-V xStAx-VHLL. **b** HEK293T cells transfected with Topflash luciferase were treated with Wnt3a CM and xStAx (xS) or xStAx-VHLL (xS-V) at indicated concentration for 24 h and then harvested for luciferase activity measurement. **c** HEK293T cells transfected with Topflash luciferase were treated with Wnt3a CM and 10 μM XAV939 (XAV), 20 μM PRI-724 (PRI), 100 μM PNU-74654 (PNU), 10 μM MSAB or 70 μM xStAx-VHLL (xS-V) for 24 h and then harvested for luciferase activity measurement. **d** HEK293T cells were treated with MSAB (10 μM) or xStAx-VHLL (70 μM) and Wnt3a CM for 24 h and then harvested for immunoblotting. **e**–**g** HCT116 (**e**), SW480 (**f**), and LoVo (**g**) cells were treated with MSAB (10 μM) or xStAx-VHLL (50 μM) for 24 h and then harvested for immunoblotting. **h** Equal amount (1 × 10^4^) of LoVo cells was treated with the vehicle DMSO, 10 μM MSAB or 50 μM xStAx-VHLL for 6 days, and the cell number was counted each day. **i** HEK293T cells were treated with MSAB (10 μM) xStAx-VHLL (70 μM) for 24 h and then replaced with fresh medium for 24 or 48 h to wash out the drugs before harvested for immunoblotting. The relative band intensity was quantified with ImageJ and normalized to GAPDH. Data from three independent experiments are displayed as the mean ± SD by two-way ANOVA (**a**, **b**, **c**, **h**) or one-way ANOVA (**d**–**g**). *P < 0.05, **P < 0.01.

We then examined the effect of xStAx-VHLL on Wnt/β-catenin signaling using the Topflash-luciferase reporter in HEK293T cells. Wnt3a induced the expression of the reporter, and SAHPA1 slightly enhanced the Wnt3a effect while xStAx exhibited an inhibitory effect (Fig. 2a), as previously reported^14^. Importantly, xStAx-VHLL was more potent in decreasing the Wnt3a-induced expression of the reporter, whereas SAHPA1-VHLL still exhibited moderate enhancement of Wnt signaling. Furthermore, xStAx-VHLL showed a dose-dependent effect on Wnt signaling (Fig. 2b).

A number of small-molecule inhibitors have been reported to target β-catenin, including PRI-724 that disrupts the β-catenin-CBP interaction^7^, PNU-74654 that disrupts the β-catenin/TCF interaction^7^ and MSAB that promotes β-catenin degradation^9^. We compared the inhibitory effect of xStAx-VHLL with those inhibitors as well as XAV939 that inhibits tankyrases and thus stabilizes Axin^23,24^. As shown in Fig. 2c, xStAx-VHLL (70 μM) exhibited a comparable inhibition on Wnt-induced Topflash-luciferase to other inhibitors at the working concentration (10 μM). Of note, higher concentration of small-molecule inhibitors caused cytotoxicity. As MSAB promotes β-catenin degradation like xStAx-VHLL, we further compared its effect on β-catenin in several cell lines. Both 10 μM MSAB and 50 μM xStAx-VHLL decreased β-catenin protein to a comparable level after 24 h treatment in HEK293T, HCT116, and SW480 cells (Fig. 2d–f). Interestingly, in LoVo cells, only xStAx-VHLL was able to reduce the β-catenin protein level, while MSAB had no effect (Fig. 2g). Consistently, the proliferation of LoVo cells was hampered by xStAx-VHLL but not by MSAB (Fig. 2h). In addition, xStAx-VHLL induced the durable degradation of β-catenin in HEK293T cells as long as 24 h post washout, while β-catenin protein level was restored after 24 h of MSAB removal (Fig. 2i). Together, these results suggest that xStAx-VHLL is a potent β-catenin degrader.

### xStAx-VHLL restrains cell proliferation and tumor formation of colorectal cancer cancers

**Fig. 3:**
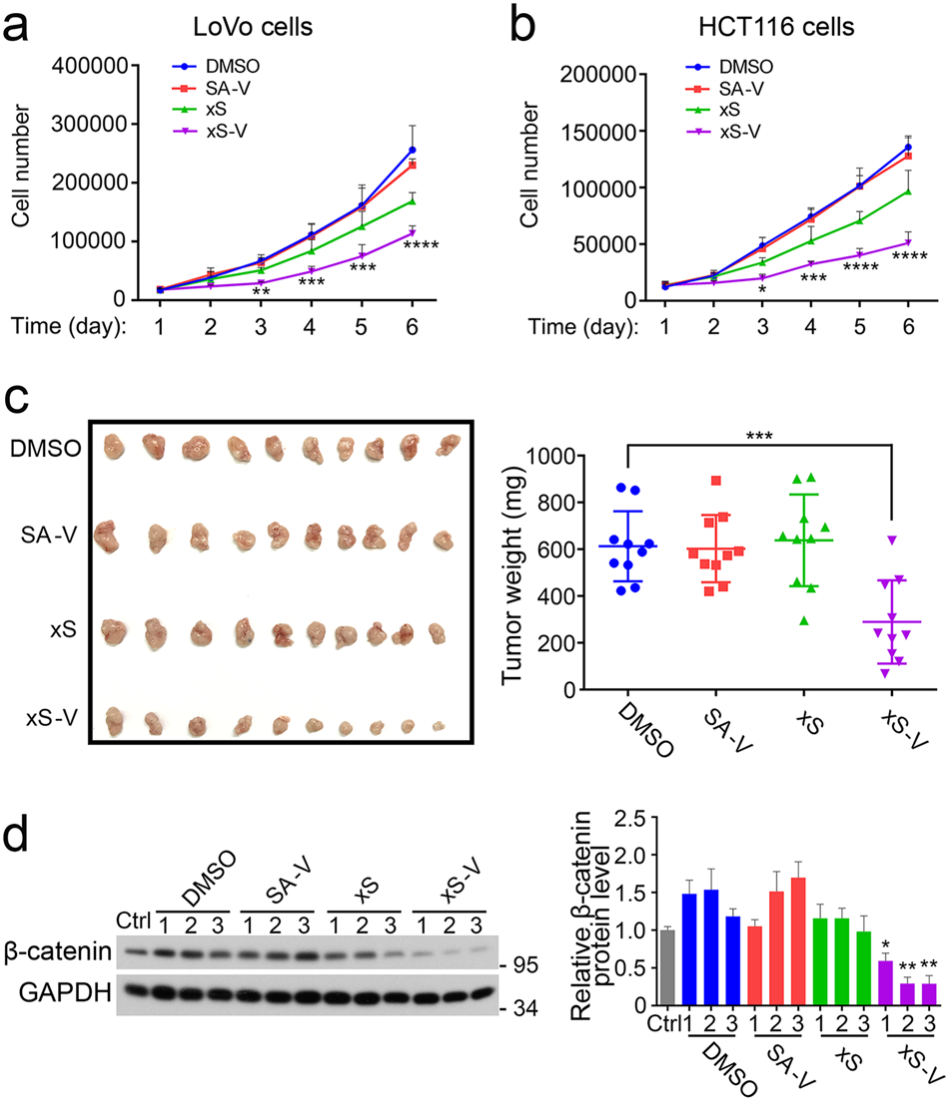
xStAx-VHLL impedes colorectal cancer cell proliferation and restrains tumor formation in nude mice. **a**, **b** Equal amount of LoVo cells (**a**) and HCT116 cells (**b**) were treated with the vehicle DMSO or 50 μM peptides at for 6 days, and the cell number was counted each day. SA-V SAHPA1-VHLL, xS xStAx, xS-V xStAx-VHLL. **c** BALB/C nude mice were subcutaneously injected with 3 × 10^6^ LoVo cells pretreated with DMSO or 50 μM peptides as indicated. After 3 weeks, the mice were sacrificed. Tumor weight was shown in the right. *n* = 10 mice per group. **d** Tumor samples from **c** were homogenized for immunoblotting. Relative β-catenin protein level was quantified. The relative band intensity was quantified with ImageJ and normalized to GAPDH levels. Data from three tumor samples are displayed as the mean ± SD by two-way ANOVA (**a**, **b**) or one-way ANOVA (**c**, **d**). *P < 0.05, **P < 0.01, ***P < 0.001.

As xStAx-VHLL impairs Wnt signaling by promoting β-catenin degradation, we examined its effect on cell proliferation. As illustrated in Fig. 3a, b, both xStAx-VHLL significantly attenuated the proliferation of LoVo and HCT116 cells.

Next, to examine the influence of the peptides on tumor formation, 3 × 10^6^ LoVo cells were pretreated with each peptide at 50 μM for 24 h, followed by subcutaneous injection into BALB/C nude mice. After 3 weeks when tumors were visible, the mice were sacrificed for comparison of subcutaneous tumors among different groups. Of note, the tumors originated from xStAx-VHLL pretreating cells were significantly smaller in size and weight (Fig. 3c). Consistently, immunoblotting analysis of the tumor extracts showed that the β-catenin protein level in the xStAx-VHLL group was apparently decreased compared with other groups, indicating that xStAx-VHLL achieved effective and durable degradation of β-catenin (Fig. 3d). Furthermore, the Wnt target genes *Axin2*, *Cyclin D1* and *Myc* were also decreased in the tumors derived from xStAx-VHLL-treated LoVo cells (Supplementary Fig. S6). These data suggest that xStAx-VHLL impedes CRC cells proliferation and restrains tumor formation.

### xStAx-VHLL inhibits tumor growth in APC^min/+^ mouse model

**Fig. 4:**
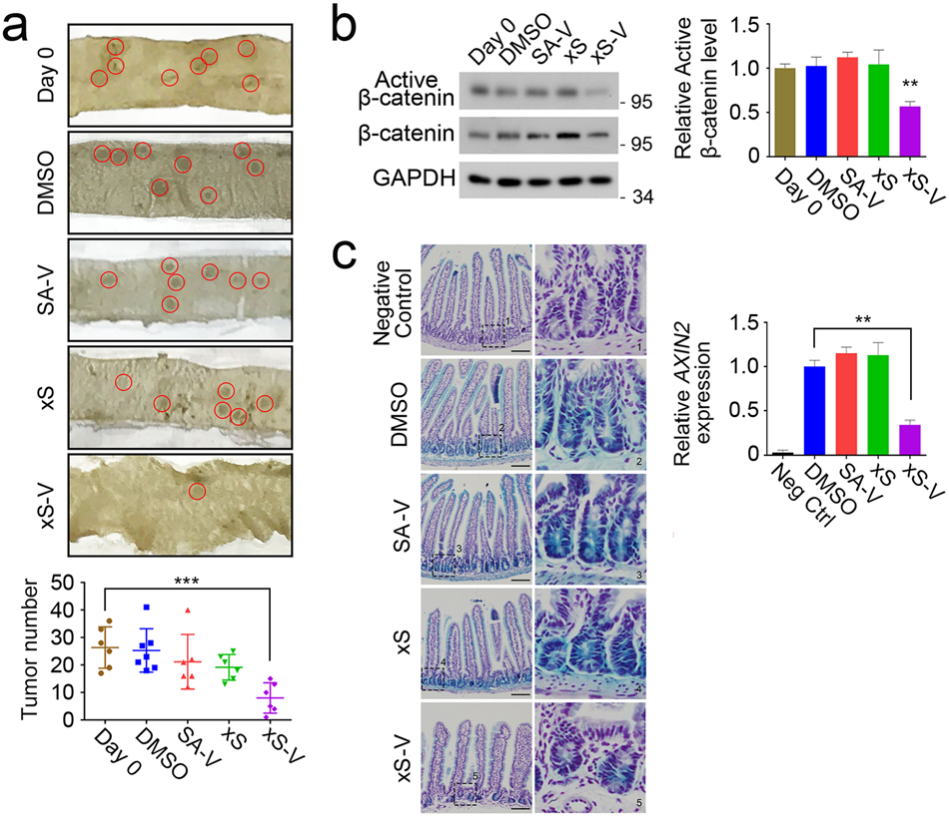
xStAx-VHLL reduces the number of intestinal tumors in *APC*^*min/+*^ mice. **a** Eleven weeks-old *APC*^*min/+*^ mice (Day 0) were intraperitoneally injected with DMSO or peptides (30 mg/kg) for 7 times at every other day (*n* = 6, 7, 6, 6, 6 mice for each group, respectively). Red circles indicate tumors. Tumor number of the whole intestinal tract was analyzed in the lower panel. SA-V SAHPA1-VHLL, xS xStAx, xS-V xStAx-VHLL. **b** The tissue of the *APC*^*min/+*^ mice small intestine was homogenized and total proteins were extracted for immunoblotting. Right panel is the quantitation. **c** The *AXIN2*-lacZ Wnt reporter mice were intraperitoneally injected with peptides for seven times at very other day. Then the mice were sacrificed for β-galactosidase staining in intestine. Relative β-galactosidase intensity was quantified in the lower panel. Scale bar = 100 μm. Data are displayed as the mean ± SD by one-way ANOVA. *P < 0.05, **P < 0.01, ***P < 0.001.

To further explore whether the PROTAC xStAx-VHLL peptide can eliminate the existing tumors, we took use of *APC*^*min/+*^ mice that harbored a point mutation in the *APC* gene, resulting in *APC* deletion and constitutive activation of Wnt signaling^25^. Because *APC*^*min*/+^ mice can develop intestinal tumors around 10 weeks-old^26^, the 11 weeks-old *APC*^*min*/+^ mice were intraperitoneally injected with 30 mg/kg vehicle DMSO or peptides at every other day for 14 days. Then the mice were sacrificed for analysis of intestinal tumors. Although tumor number was slightly decreased in the SAHPA1-VHLL and xStAx groups, the xStAx-VHLL dramatically reduced tumor number compared with that of 11 weeks-old mice before treatment (day 0) or with vehicle DMSO treatment (Fig. 4a). In agreement with it, the active of β-catenin protein levels were also decreased compared with the vehicle group (Fig. 4b). To further confirm the in vivo activity of xStAx-VHLL, we used *Axin2*^*LacZ*^ Wnt reporter mice to examine *Axin2* expression. After intraperitoneal injection of the peptides for 7 doses/14 days, the mice were sacrificed and small intestines prepared for in situ β-galactosidase staining. xStAx-VHLL significantly blocked *Axin2* expression (Fig. 4c). Taken together, these data indicate that xStAx-VHLL is an effective reagent to decrease the existing tumors.

### xStAx-VHLL impedes the growth of wild-type and APC^−/−^ organoids

**Fig. 5:**
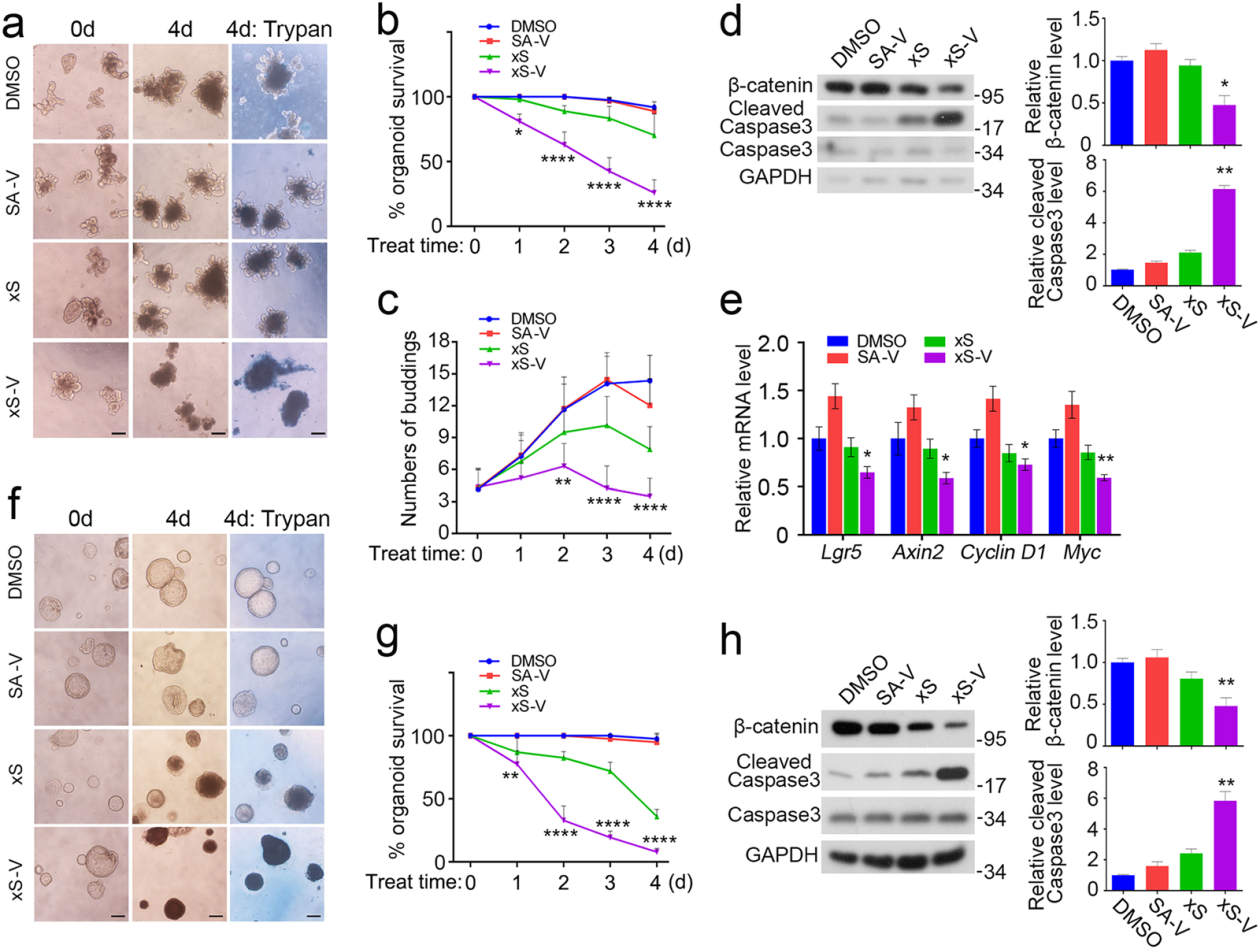
xStAx-VHLL inhibits the growth of wild-type and *APC*^−/−^ organoids. **a** Wild-type organoids were cultured with 10 μM peptides for 4 days. The trypan blue staining at day 4 indicated the dead organoids. Scale bar = 100 μm. SA-V SAHPA1-VHLL, xS xStAx, xS-V: xStAx-VHLL. **b** The percentage of survived wild-type organoids was calculated each day with 10 μM peptides treatment. **c** The budding number of wild-type organoids was counted for each group every day. **d** Wild-type organoids were cultured with 10 μM peptides for 24 h and then harvested for immunoblotting. Relative β-catenin and cleaved caspase3 protein levels were quantified. **e** Wild-type organoids were cultured with 10 μM peptides for 3 h and harvested for qPCR analysis of Wnt target genes. **f** *APC*^−/−^ organoids were cultured with 50 μM peptides for 4 days. The trypan blue staining at day 4 indicated the dead organoids. Scale bar = 100 μm. SA-V SAHPA1-VHLL, xS xStAx; xS-V xStAx-VHLL. **g** The percentage of survived *APC*^–/–^ organoids was calculated each day with 10 μM peptides treatment. **h** *APC*^–/–^ organoids were cultured with 50 μM peptides for 24 h and then harvested for immunoblotting. Relative β-catenin and cleaved caspase3 protein levels were quantified. The relative band intensity was quantified with ImageJ and normalized to GAPDH levels. Data from three independent experiments are displayed as the mean ± SD by two-way ANOVA (**b**, **c**, **e**, **g**) or one-way ANOVA (**d**, **h**). *P < 0.05, **P < 0.01, ***P < 0.001.

In the past few years, intestinal organoids have become an ideal *ex vivo* culture system to screen the drugs that influence the signaling pathways involved in organoids survival and growth and treat cancer^26^. As Wnt/β-catenin signaling is essential to maintain intestinal organoid growth^26,27^, we then tested whether xStAx-VHLL could impede the growth of intestinal organoids by achieving effective β-catenin degradation. Firstly, we cultured the wild-type organoids with peptides at 10 μM for 4 days (Fig. 5a). xStAx-VHLL treatment obviously impeded organoid survival and bud formation, and this inhibitory effect was observed after 1 day treatment (Fig. 5a–c). Consistently, xStAx-VHLL treatment reduced the β-catenin protein level while increased the cleaved caspase 3 level (Fig. 5d). The Wnt signaling target genes *Lgr5*, *Axin2*, *Cyclin D1*, and *Myc* were remarkably decreased as soon as 3 h treatment of xStAx-VHLL (Fig. 5e). Next, we chose the GFP-marked Lgr5^+^ organoids to observe the intestinal stem cell population^28^. As shown in Supplementary Fig. S7, Lgr5^+^ intestinal stem cells were reduced after 36 h treatment of xStAx-VHLL, and the viable cell population also decreased, consistent with the above caspase 3 activation.

We also tested the effect of xStAx-VHLL on the *APC*^−/−^ organoids. The treatment of xStAx-VHLL (50 μM) significantly impeded the growth of *APC*^−/−^ organoids, and the inhibitory effect was observed at day 3 (Fig. 5f, g). As shown in Fig. 5h, immunoblotting analysis showed that β-catenin protein level in of the organoids was greatly decreased after 24 h treatment of xStAx-VHLL, whereas the cleaved caspase 3 increased. xStAx-VHLL achieved a strong inhibition on *APC*^*−/−*^ organoid survival, whereas MSAB had a minimal effect (Supplementary Fig. S8a). Consistently, xStAx-VHLL also downregulated the expression of the Wnt target genes *Axin2*, *Cyclin D1*, and *Myc* in *APC*^*−/−*^ organoids (Supplementary Fig. S8b). These results together suggest that the PROTAC peptide xStAx-VHLL exhibits strong inhibitory effects on Wnt/β-catenin signaling in wild-type and *APC*^-/-^ organoids.

### xStAx-VHLL restrains the survival of CRC patient-derived organoids

**Fig. 6:**
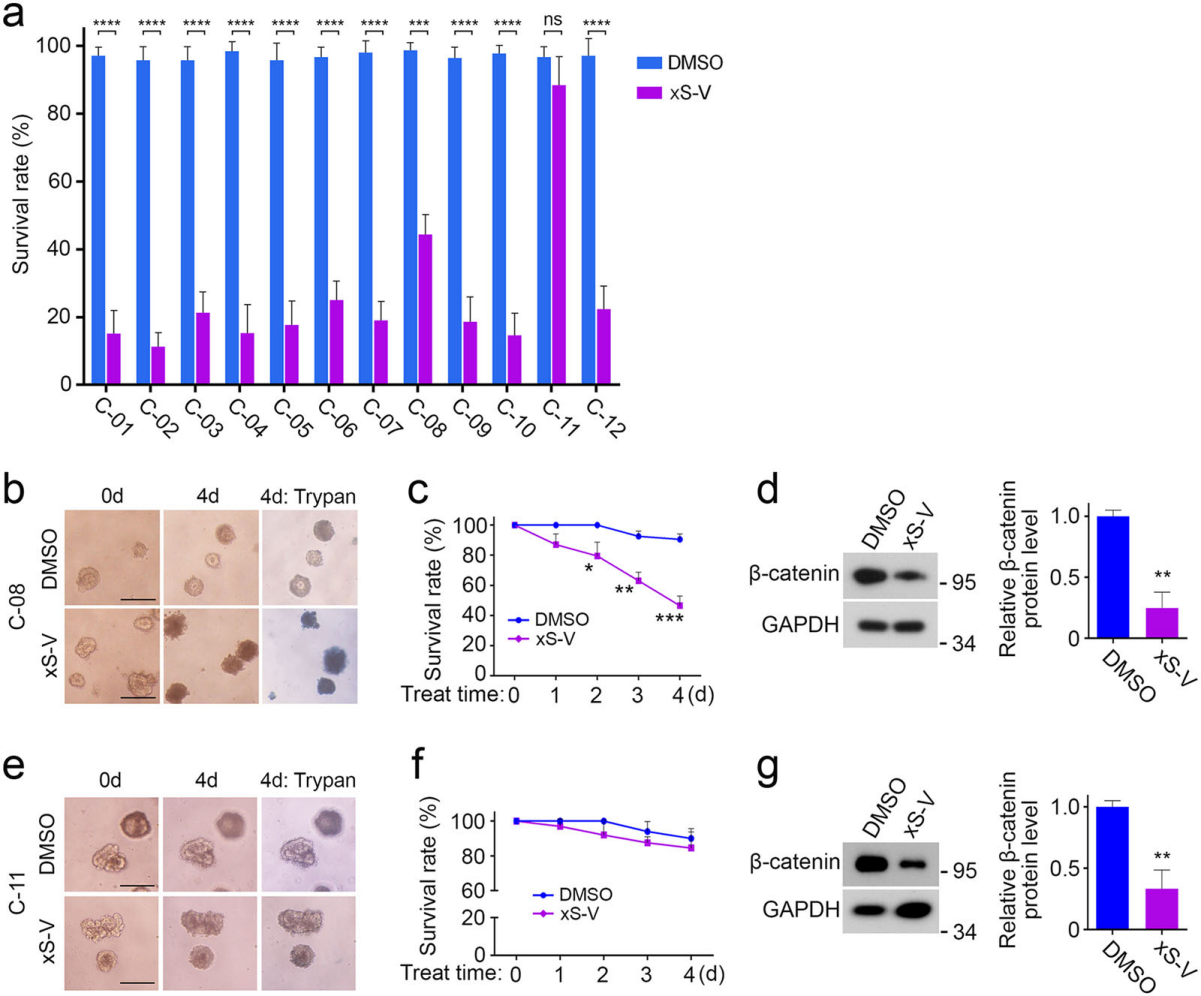
xStAx-VHLL restrains the survival of CRC patient-derived organoids. **a** Twelve CRC patient-derived organoids, numbered from C-01 to C-12, were treated with DMSO and 10 μM xStAx-VHLL for 4 days. The organoids survival rate of each group was calculated at day 4. **b** The C-08 organoids were treated with DMSO and 10 μM xStAx-VHLL for 4 days. The trypan blue staining at day 4 indicated the dead organoids. Scale bar = 100 μm. **c** The percentage of survived C-08 organoids was calculated each day after xStAx-VHLL treatment. **d** The C-08 organoids were treated with DMSO or 10 μM xStAx-VHLL for 48 h and then harvested for immunoblotting. Relative β-catenin protein levels were quantified. **e** The C-08 organoids were treated with DMSO and 10 μM xStAx-VHLL for 4 days. Scale bar = 100 μm. **f** The percentage of survived organoids in C-11 group was calculated each day after xStAx-VHLL treatment. **g** The C-11 organoids were treated with DMSO or 10 μM xStAx-VHLL for 48 h and then harvested for immunoblotting. Relative β-catenin protein levels were quantified. The relative band intensity was quantified with ImageJ and normalized to GAPDH levels. Data from three independent experiments are displayed as the mean ± SD by two-way ANOVA (**a**, **c**) and one-way ANOVA (**d**, **g**). *P < 0.05, **P < 0.01, ***P < 0.001, ****P < 0.0001.

In the majority of CRC patients, Wnt/β-catenin signaling is aberrantly activated. Owing to the successful 3D culture of colorectal cancer tissue derived organoids, we were able to test the clinical potential of β-catenin targeted degrader xStAx-VHLL *ex vivo*. We collected 12 CRC samples (numbered from C-01 to C-12). The exon sequencing indicated the mutations of Wnt/β-catenin pathway components in all tumor tissue (Supplementary Fig. S9). As shown in Fig. 6a, 10 μM xStAx-VHLL could potently inhibited the survival of the 11/12 patient-derived organoids in 4 days, including C-05 that harbored multiple mutations of *LRP6, APC, AXIN2, CTNNB1* (β-catenin), *TCF7L2, KRAS, TP53, SMAD4, PI3KCA*, and others. For example, xStAx-VHLL manifested a strong inhibition function on C-08 and C-05 compared with the DMSO control (Fig. 6b, c and Supplementary Fig. S10). Consistently, xStAx-VHLL reduced the β-catenin protein level after 2 days treatment (Fig. 6d). However, C-11 organoids, which harbored the mutations of *LRP6, APC*, *TCF7L2, KRAS, TP53*, and others, were insensitive to xStAx-VHLL, even though the β-catenin protein level was decreased (Fig. 6e–g). It is possible that other signaling, rather than Wnt/β-catenin signaling, plays a major role in driving the growth and survival of C-11 organoids.

## Discussion

As Wnt/β-catenin signaling pathway is activated in various cancers, including colorectal cancer, targeting β-catenin has been regarded as an important approach to treat these diseases^4,5^. In this study, we generated two PROTAC peptides by coupling VHL ligand to the stapled peptides that have been shown to interact with β-catenin^13,14^: SAHPA1-VHLL and xStAx-VHLL. We demonstrated that xStAx-VHLL acted as a potent PROTAC β-catenin degrader, efficiently promoting the proteasomal degradation of endogenous β-catenin in cancer cells and intestinal organoids. xStAx-VHLL also efficiently inhibited Wnt signaling as well as cell proliferation, intestinal organoid growth, and tumor formation. This inhibitory effect was observed not only in the systems with intact Wnt/β-catenin signaling, but also in the systems with APC-defective and constitutively active β-catenin mutations. Further, in CRC patient-derived tumor organoids, xStAx-VHLL effectively restrained the survival of tumor organoids, which highlighted its clinical potential.

In the past few years, several small molecules have been identified to target β-catenin. It has been shown that MSAB interacts with β-catenin and then induces the proteasomal degradation of β-catenin^9^, and NRX promotes the β-catenin degradation by enhancing the interaction between β-catenin and the ubiquitin E3 ligase βTrCP^29^. PRI-724, PNU-74654, and BC2059 are capable of disrupting the interactions between β-catenin and its co-activators^7^. However, the specificity of small molecules is a general concern.

Mimicking the β-catenin binding region of Axin, we and Verdine lab have independently designed stapled helical peptides^13,14^. Although xStAx designed by Grossmann and coworkers exhibited an impressive inhibition on Wnt/β-catenin signaling, it could be less efficient for in vivo use to treat tumors as the newly synthesized β-catenin can rapidly accumulate^30^. The PROTAC technique could aid small molecule inhibitors or peptides to exert better biological activities in vivo as PROTAC can link the ubiquitin-proteasome system to induce effective degradation of targeted proteins^16^. Indeed, xStAx-VHLL was more potent than xStAx to inhibit Wnt/β-catenin signaling and attenuate cell proliferation and organoid growth. More importantly, xStAx-VHLL was able to impede in vivo tumor formation in nude mouse xenografts and suppress the existing tumors in *APC*^*min*/+^ mouse models, while xStAx had minimal effect in vivo.

Traditional drug discovery heavily relies on cell-based or animal models, few of the candidate drugs succeed in patient-based clinical studies. The patient-derived tumor organoids, which recapitulate the complexity and heterogeneity of tumors, have been shown to be an ideal model for drug screening and precision medicine^26^. In this study, we have found that a potent inhibition of 11/12 patient-derived tumor organoids by xStAx-VHLL not only reveals that xStAx-VHLL is a promising anti-CRC drug candidate, but also confirms that the patient-derived tumor organoids exhibit their advances in testing the effectiveness of PROTAC peptides.

Although both SAHPA1 and xStAx were derived the same β-catenin-binding peptide, unlike xStAx-VHLL, SAHPA1-VHLL had minimal effect on β-catenin stability and thus no much effect on Wnt/β-catenin signaling. It could be due to the promoting effect of SAHPA-1^13^ and the lower membrane permeability of SAHPA1-VHLL compared to xStAx-VHLL.

There is more space for the improvement of the working efficacy of xStAx-VHLL. The VHL peptide ligand inserted in this study consists of natural amino acids, which could be degraded once the PROTAC peptide enters the cell. Non-natural residues substitution instead of original ones, such as reported hydroxyproline to replace regular proline^31,32^, could be an effective approach to enhance the proteolytic resistance of PROTAC. It was previously reported that peptide double-stapling at both N-terminals and C-terminals rather than single stapling conferred striking protease resistance upon the lengthy peptide^33^. Moreover, Replacement of the VHL ligand with other E3 ligands is worthy to be tested. Improvement of β-catenin-binding peptide could also be another direction. Grossmann and coworkers reported the optimization of xStAx through homo-Arg replacement and N-terminal polar modification, which yielded NLS-StAx-h with highly membrane permeability^34^. Whether NLS-StAx-h is a better β-catenin ligand to design PROTAC may be of interest. Last, optimization of the chemical linker is needed. The simple Ahx linker is a little bit short and more polar PEG linker bearing varying length should be taken into consideration^35^. Nonetheless, as the first-generation PROTAC β-catenin degrader, xStAx-VHLL has already showed great promise in inhibiting tumor growth in vivo, and it could be employed as the leading PROTAC for further optimization in Wnt signaling-interfering cancer therapy.

## Materials and methods

### Chemical synthesis and characterization of PROTACs

All reagents were purchased from Adamas-beta®, J&K Scientific, GL Biotech, Energy Chemical or Sinopharm Chemical Reagent Co. Ltd. Rink Amide MBHA resin (0.30 mmol/g loading) was purchased from Tianjin Nankai Hecheng Science & Technology Co. Ltd.

As a typical example, 400 mg Rink Amide MBHA resin was swelled with DCM (5 mL) for 20 min. Then the resin was treated with 20% piperidine/DMF/0.1 mol/L Oxyma pure twice (5 min × 2), followed by washing with DMF (five times), DCM (five times) and DMF (five times) at 35 °C. For coupling of the natural amino acid, Fmoc-AA-OH (1 mmol), DIC (1 mmol), Oxyma pure (1 mmol) and NMP (6 mL) were mixed for 1 min and then added to the resin at 60 °C. After 15 min, the resin was washed with DMF (five times), DCM (five times), and DMF (five times). The peptide couplings of S5 and R8 were carried out over a single 2 h coupling cycle using 2 eq. of the Fmoc protected amino acids at 60 °C. The deprotection, washing, coupling and washing steps were repeated until all the amino acid residues were assembled reagent. The peptide-bound resin was treated with 20% piperidine/DMF/0.1 mol/L Oxyma pure to remove the Fmoc group from the N-terminus. After the resin was washed, it was treated with 5 mL solution of acetic anhydride and pyridine (1:1) for 20 min. Then the resin was washed with DMF (five times), DCM (five times), and DMF (five times). The ring-closing metathesis reaction was carried out in 1,2-dichloroethane (DCE) at room temperature using Grubbs’ first-generation catalyst. The resin was washed with DCM (three times), and DCE (three times) and then treated with 10 mM solution of Grubbs’ first-generation catalyst in DCE. After the first round of the 2 h metathesis, we repeated the same procedure for a second round of catalyst treatment with fresh catalyst solution. Then the peptide-resin was washed with DMF (five times) and DCM (five times). Peptides were cleaved from their resin by treatment with cocktail trifluoroacetic acid (TFA) (TFA/Triisopropylsilane (TIPS)/H_2_O = 95/2.5/2.5, v/v/v) for 2 h at room temperature. After completion of the cleavage reaction, TFA was evaporated by blowing with Argon. The crude peptides were obtained by precipitation with 40 mL of cold diethyl ether and centrifugation at 3500 r/min for 3 mins (three times). The supernatant diethyl ether was decanted from the centrifuge tube, the crude peptides were allowed to air dry and purified by RP-HPLC to give the final products.

After elongation of the peptide and metathesis reaction were conducted on the resin according to a general protocol. Then, the Fmoc group was removed and the resin was treated with Fmoc-Ahx-OH (4 eq.), DIC (4 eq.), Oxyma pure (4 eq.) and NMP (6 mL) for 15 min at 60 °C. The Fmoc group was removed and the resin was treated with fluorescein isothiocyanate (FITC, 2 eq.) and DIPEA (2 eq.) in DMF (6 mL) at room temperature overnight. The biotinylated labeling was carried out as routine amino acid. Cleavage was conducted following a general protocol and crude peptide was purified by RP- HPLC to give the final products.

### Antibodies, reagents, and cell lines

Antibodies used in this study were as following: β-catenin and caspase3 were purchased from Santa Cruz; GAPDH, cleaved caspase3, Axin1, Dvl2, GSK3β; TCF4 from Cell Signaling Technology and E-cadherin from BD Biosciences. XAV939 and VH-298 was purchased from MCE, MSAB from Sigma, PRI-724 and PNU-74654 from Selleck. Peptides used in this study, including SAHPA1, SAHPA1-VHLL, xStAx and xStAx-VHLL, were dissolved in DMSO for 10 mM stock solution.

Colorectal cancer cell lines SW480, HCT116, and LoVo were obtained from ATCC. SW480 cells were cultured in L15 medium supplemented with 10% FBS (Hyclone) in a 37 °C humidified incubator without CO_2_. HCT116 cells were cultured in McCoy’s 5 A medium supplemented with 10% FBS (Hyclone) in a 37 °C humidified incubator containing 5% CO_2_. LoVo cells were cultured in DMEM medium supplemented with 10% FBS (Hyclone) in a 37 °C humidified incubator containing 5% CO_2_.

### Topflash luciferase reporter assay

Dual luciferase reporter assay system (Promega) was used. HEK293T cells, cultured in 24-well plates, were transfected with 200 ng Topflash plasmid and 30 ng Renilla plasmid for 3-well cells. At 12 h post-transfection, cells were treated with certain concentrations of peptides with or without Wnt3a conditioned medium (CM) for another 24 h. Then harvested the cells for luciferase activity measurement.

### Immunoprecipitation and immunoblotting

For immunoprecipitation, the cells were lysed in TNE lysis buffer (50 mM Tris-HCl, pH 8.0, 150 mM NaCl, 1% Nonidet P-40, and protease inhibitor cocktail). For the β-catenin ubiquitination detection experiment, the raw cell lysates were heated to 95 °C for 5 min and sonicated to completely denature the proteins. After 18,000×*g* centrifugation for 10 min, the lysates were immunoprecipitated with specific antibody and protein A-Sepharose beads and then washed the beads with TNE buffer for three times before immunoblotting.

### Mouse intestine organoid culture

Mouse intestinal crypts isolation and organoid culture were performed as previously described^36^. Briefly, mouse intestine was cut longitudinally and washed three times with cold PBS. Villi were carefully scraped away and small pieces (5 mm) of intestine were incubated in 0.5 M EDTA in PBS for 40 min on ice. These pieces were then vigorously suspended in cold PBS and the mixture was passed through 70 μm cell strainer (BD Biosciences). The crypt fraction was enriched through centrifugation (3 min at 300–400 g). Then the crypts were embedded in Matrigel (BD Biosciences) and seeded on 48-well or 24-well plates. The intestinal organoid culture medium consists of Advanced DMEM/F12 supplemented with Penicillin/Streptomycin, GlutaMAX-I, N2, B27 and N-acetylcysteine (Sigma-Aldrich) and combination of growth factors including EGF (50 ng/mL, Invitrogen), Noggin (100 ng/mL, R&D) and R-spondin1 (500 ng/mL). The organoids were treated with proper dilution of peptides in the medium.

### CRC patient tumor tissue collection and ethics statement

Intestine tumor tissues were surgically resected and sampled, from 12 patients who had been diagnosed with intestine tumors at Peking University Third Hospital, Beijing, China, as referred before^37^. All samples were obtained with informed consent, and the study was approved by the Peking University Third Hospital Medical Science Research Ethics Committee (M2018083). All relevant ethical regulations of Peking University Third Hospital Medical Science Research Ethics Committee were followed.

### Patient-derived tumor organoid culture

CRC patient tumor tissues were washed with cold HBSS. After removing the muscle tissue, the epithelial tumor tissues were cut into small pieces and vigorously suspended. Then the tissue suspension was centrifuged (3 min at 300–400 g) and tumor fraction was enriched at the bottom. Next, the tumor fraction was embedded in Matrigel (BD Biosciences) and seeded on 24-well plates with the culture medium, which includes Advanced DMEM/F12 supplemented with Penicillin/Streptomycin, GlutaMAX-I, N2, B27, N-acetylcysteine (Sigma-Aldrich), CHIR-99021 (5 μM, Selleck), A-83–01 (0.5 μM, Cayman), SB202190 (10 μM, Selleck), gastrin (1 nM, Tocris), Y27632 (10 μM, Enzo), PEG2 (2.5 μM, Selleck), nicotinamide (10 mM, Sigma-Aldrich), EGF (50 ng/mL, Invitrogen), Noggin (100 ng/mL, R&D) and R-spondin1 (500 ng/mL).

### Quantitative RT-PCR

Total RNA was extracted with TRIzol (Invitrogen) and cDNA was prepared using Revertra Ace (Toyobo). Real-time PCR reactions were performed in triplicates on a LightCycler 480 (Roche). Relative target genes expression values were normalized to GAPDH expression. Data were analyzed according to the ΔCT method. The following primers were used: *Lgr5*, 5′-CGGGACCTTGAAGATTTCCT-3′ and 5′-GATTCGGATCAGCCAGCTAC-3′; *Aixn2*, 5′-GCTCCAGAAGATCACAAAGAGC-3′ and 5′-AGCTTTGAGCCTTCAGCATC-3′; *Ccnd1*, 5′-GCCATCCAAACTGAGGAAAA-3′ and 5′-GATCCTGGGAGTCATCGGTA-3′; *Myc*, 5′- GCTGTTTGAAGGCTGGATTTC-3′ and 5′-GATGAAATAGGGCTGTACGGAG-3′; and *Gapdh*, 5′-AAGAAGGTGGTGAAGCAG-3′ and 5′-TCATACCAGGAAATGAGC-3′.

### Cell proliferation assay

Equal number of cells (8000–10,000 cells) were seeded in the 24-well plates and then subjected to different treatments. The cell numbers were counted every 24 h with the CCK-8 cell counting kit (Dojindo Molecular Technologies) according to manufacturer’s instructions.

### Tumor formation in nude mice

LoVo cells cultured were pretreated with DMSO pr peptides (50 μM) for 24 h. Then the cells were digested with trypsin and centrifuge (3 min at 300–400 g). Cell pellets were washed with cold PBS three times and the cell number was counted. The BALB/C nude mice were subcutaneously injected with 3 × 10^6^ cells. Three weeks later, the nude mice were sacrificed and dissected for tumors. Tumor weight was quantified.

### Immunofluorescence

The small intestines were isolated from *APC*^*min/+*^ mice and fixed in 4% paraformaldehyde at room temperature overnight. Paraffin sections (5 μm) were cut (RM2235, Leica). The sections were de-paraffinized in xylene and graded alcohols, followed by antigen retrieval in 95 °C water bath for 25 min, followed by permeabilization with 0.1% Triton X-100 for 15 min at 4 °C and block for 1 h at room temperature in 5% BSA. After incubation overnight at 4 °C with the primary antibody (rabbit anti-Ki67, Abcam, ab15580, 1:300; mouse anti-β catenin, Santa Cruz, sc7963, 1:1000), the fluorescein-labeled secondary antibody (Life Technologies, 1:300) was added for 1 h at room temperature. The sections were scanned using Olympus FV3000 Laser Scanning Microscope.

### *Axin2*^LacZ^ β-galactosidase staining

The small intestines were isolated from *Axin2*^LacZ^ reporter mice, washed with PBS and fixed in 4 % paraformaldehyde for 1 h at room temperature, then cut into small pieces (1 cm). The tissue pieces were stained with in situ β-galactosidase staining kit (Beyotime) according to the manufacturer’s instruction. The stained tissues were transferred to tissue cassettes and paraffin blocks prepared using standard methods. The tissues were sliced into 5 μm thick sections with paraffin slicing machine (RM2235, Leica) and subjected to hematoxylin staining.

### Statistical analysis

All experiments were carried out with at least three biological replicates. GraphPad Prism 6 software was employed for statistical analysis. For reporter assays, qRT-PCR, cell proliferation assays, immunofluorescence marker intensity and organoid experiment, statistical analysis was performed with two-way ANOVA. For immunoblot band intensity, tumor weight of nude mice, tumor number of *APC*^*min/+*^ mice and relative *Axin2* expression level, statistical analysis was performed with one-way ANOVA. Results were presented as mean ± SD. *P* < 0.05 was considered statistically significant: *P < 0.05, **P < 0.01, and ***P < 0.001.

## Supplementary information

Supplementary Figures

## Supplementary information

**Supplementary Information** accompanies the paper at (10.1038/s41421-020-0171-1).
