# Supplementary material for: A PROTAC peptide induces durable β-catenin degradation and suppresses Wnt-dependent intestinal cancer
Source: Cell Discov. 2020 Jun 9;6:35. doi: 10.1038/s41421-020-0171-1 (PMC7280531; doi:10.1038/s41421-020-0171-1)
Supplement: Supplementary file 1 — Supplementary Figures [file 41421_2020_171_MOESM1_ESM.pdf]

Supplementary materials

A PROTAC peptide induces durable β-catenin degradation and suppresses Wnt-dependent intestinal cancer

Hongwei Liao[1,6], Xiang Li[2,6], Lianzheng Zhao[1,6], Yalong Wang[1], Xiaodan Wang[1], Ye Wu[2], Xin Zhou[5], Wei Fu[5], Lei Liu[4], Hong-Gang Hu[2,3*] & Ye-Guang Chen[1,*]

[1]The State Key Laboratory of Membrane Biology, Tsinghua-Peking Center for Life Sciences, School of Life Sciences, Tsinghua University, Beijing 100084, China
[2]School of Pharmacy, Second Military Medical University, Shanghai 200433, China
[3]Institute of Translational Medicine, Shanghai University, Shanghai 200444, China.
[4]Tsinghua-Peking Center for Life Sciences, Department of Chemistry, Tsinghua University, Beijing 100084, China
[5]Department of General Surgery, Peking University Third Hospital, Beijing, China

[6]These authors contributed equally

Supplementary Figures 1-10

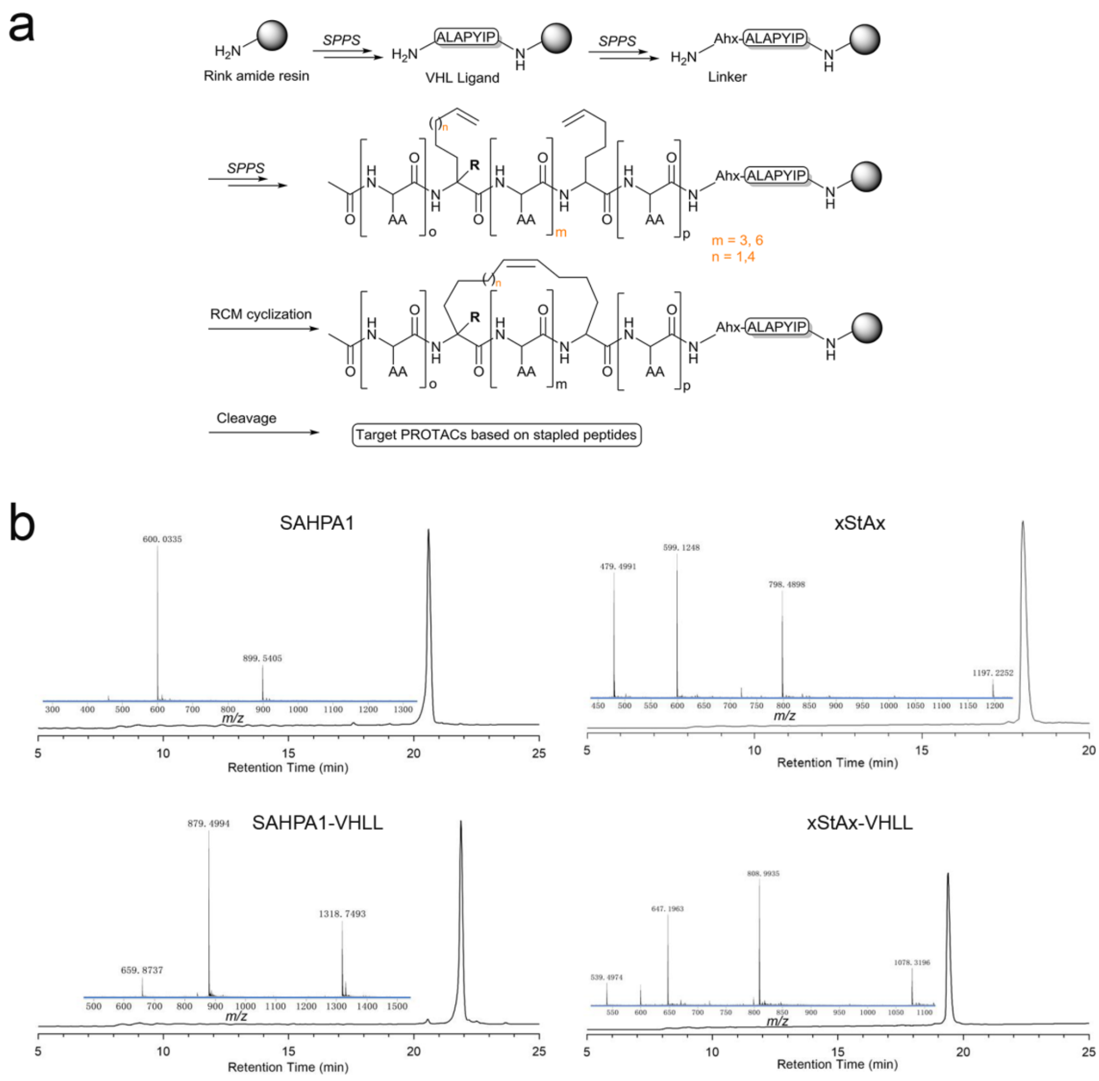

Supplementary Fig. S1. Chemical synthetic route of PROTAC peptides and their purity control.

(a) Solid phase synthesis steps of PROTACs. (b) High resolution mass spectra and HPLC trace of peptides. SAHPA1: HR-Q-TOF-MS *m/z* calcd for $C_{83}H_{141}N_{23}O_{19}S$ 1796.0495; found $[M+2H]^{2+}$ = 899.5405, $[M+3H]^{3+}$ = 600.0335. SAHPA1-VHLL: HR-Q-TOF-MS *m/z* calcd for $C_{126}H_{207}N_{31}O_{28}S$ 2634.5448; found $[M+2H]^{2+}$ = 1318.7493, $[M+3H]^{3+}$ = 879.4994, $[M+4H]^{4+}$ = 659.8737. xStAx: HR-Q-TOF-MS *m/z* calcd for $C_{111}H_{178}N_{40}O_{20}$ 2391.4141; found $[M+2H]^{2+}$ = 1197.2252, $[M+3H]^{3+}$ =

798.4898, $[M+4H]^{4+}$ = 599.1248, $[M+5H]^{5+}$ = 479.4991. xStAx-VHLL: HR-Q-TOF-MS *m/z* calcd for $C_{111}H_{178}N_{40}O_{20}$ 3229.9094; found $[M+3H]^{3+}$ = 1078.3196, $[M+4H]^{4+}$ = 808.9935, $[M+5H]^{5+}$ = 647.1963, $[M+6H]^{6+}$ = 539.4974.

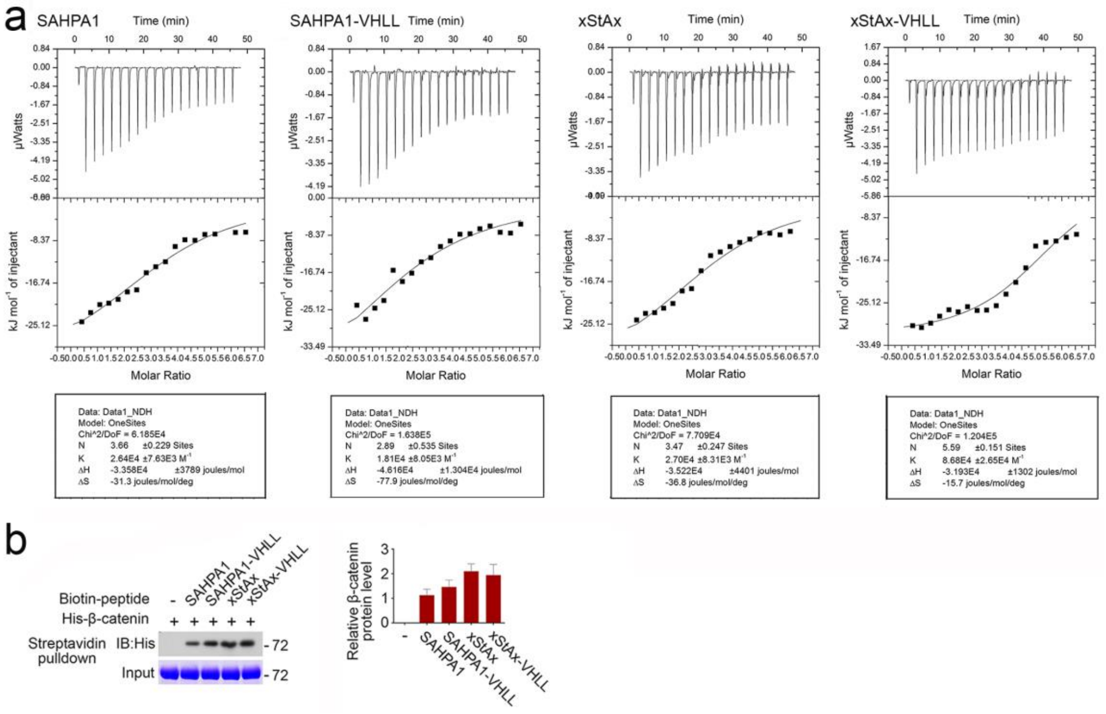

Supplementary Fig. S2. The binding ability of the peptides with purified β-catenin protein *in vitro*.

(a) Isothermal titration calorimetry (ITC) analysis to measure the interaction between peptides (1 mM) and purified β-catenin (133-665) protein (20 μM). β-Catenin (133-665) was purified from *E. coli* with His-tag. SAHPA1 Kd=38 μM; SAHPA1-VHLL Kd=55 μM; xStAx Kd=37 μM; xStAx-VHLL Kd=12 μM. (b) Biotinylated peptides were incubated with streptavidin beads and then employed to pull down purified His-β-catenin (133-665) protein. The input β-catenin protein level was stained with Coomassie brilliant blue. The pulled down β-catenin was immunoblotted with anti-His antibody, and relative band intensity was quantified.

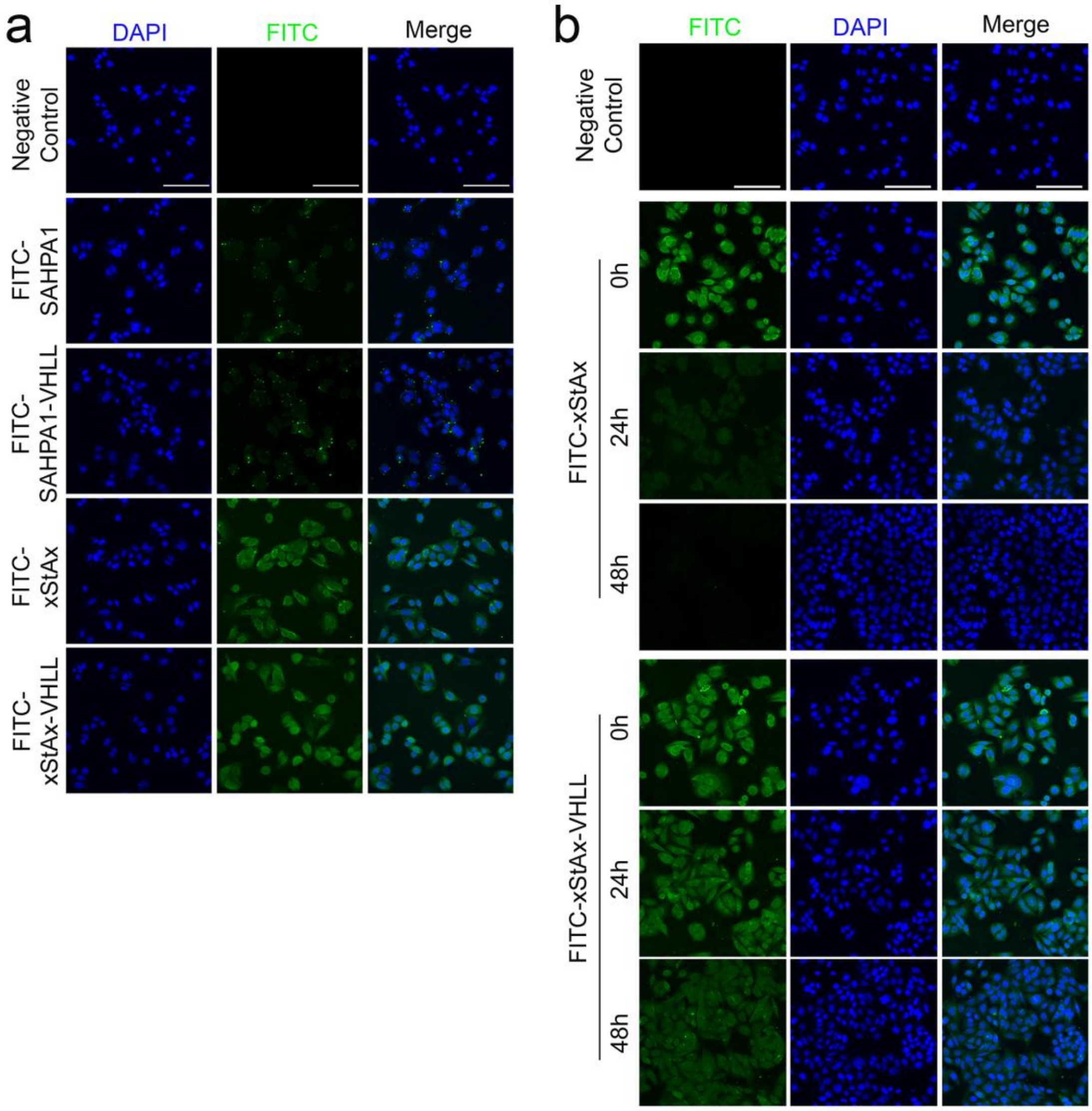

Supplementary Fig. S3. Cell permeability of FITC-labeled peptides in SW480 cells.

(a) The uptake of FITC-labeled peptides into SW480 was examined after incubation for 6 h by confocal microscopy. The nuclei were counter-stained with DAPI. Scale bar = 100 μm. (b) FITC-xStAx and FITC-xStAx-VHLL were added to SW480 cells for 6 h, and then washed out with the growth medium. After 24 h and 48 h, the cells were counter-stained with DAPI and examined by confocal microscopy. Scale bar = 100 μm.

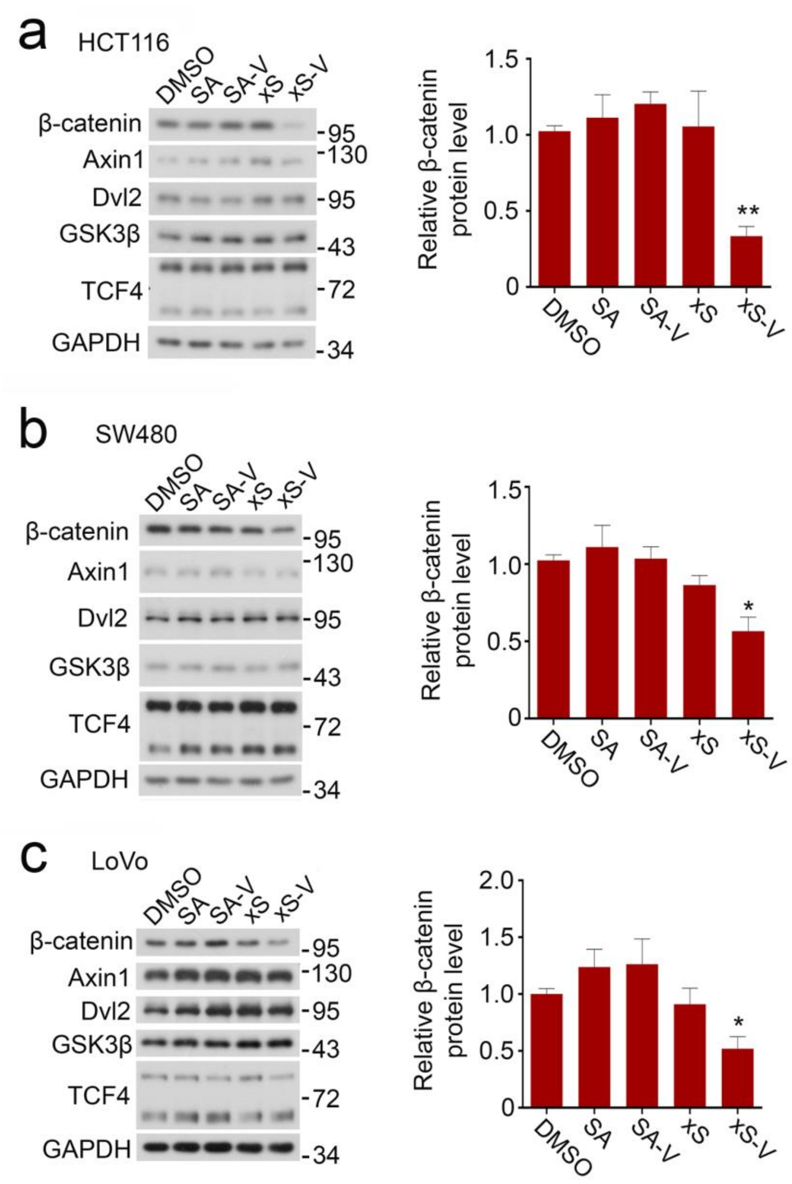

Supplementary Fig. S4. xStAx-VHLL promotes the degradation of β-catenin in colorectal cancer cells.

HCT116 (a), SW480 (b) or LoVo cells (c) were treated with 50 μM peptides for 24 h, and then harvested for immunoblotting with the antibodies as indicated. Relative β-catenin protein level was quantified with ImageJ and normalized to GAPDH. Data from 3 independent experiments are displayed as the mean ± SD by one-way ANOVA.

$^*p < 0.05$, $^{**}p < 0.01$. SA: SAHPA1; SA-V: SAHPA1-VHLL; xS: xStAx; xS-V: xStAx-VHLL.

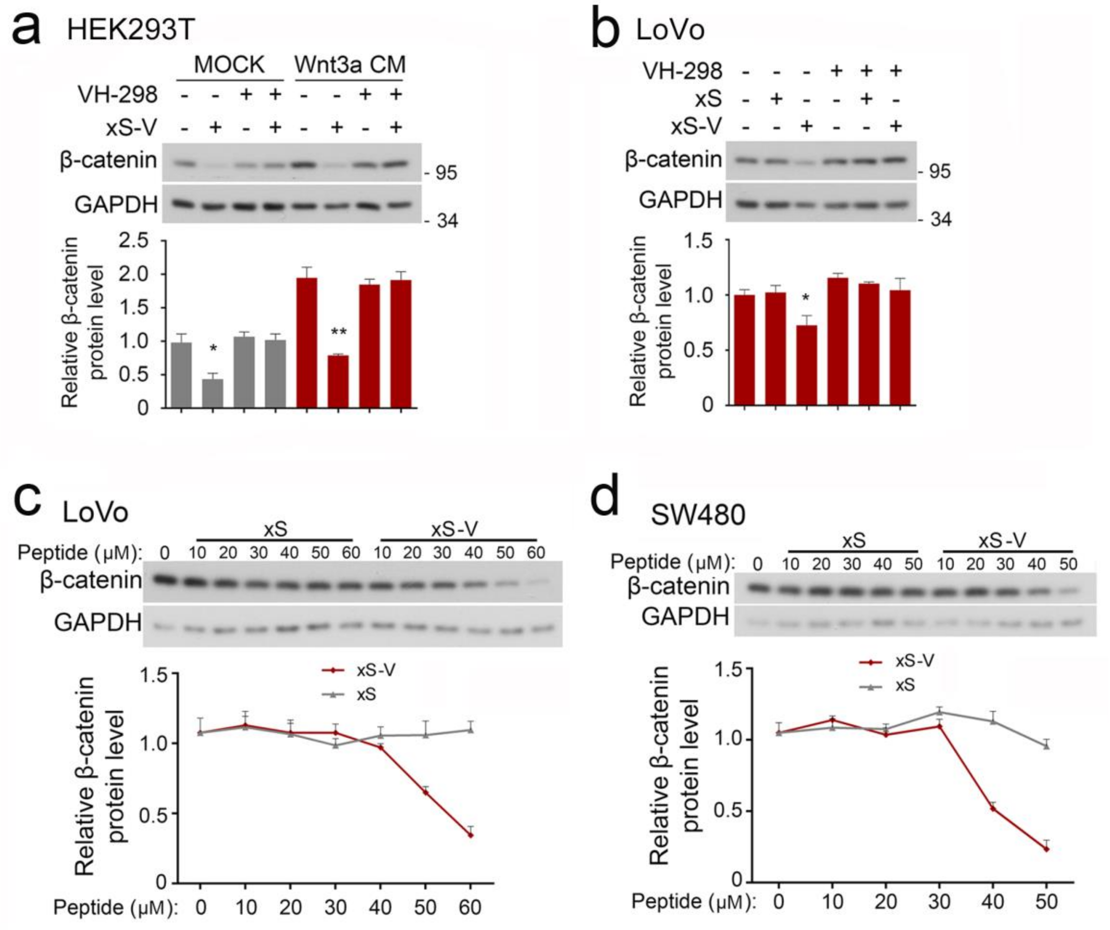

Supplementary Fig. S5. The function of xStAx-VHLL depends on the VHL ligase activity, and xStAx-VHLL reduces β-catenin protein level in colorectal cancer cells in a dose-dependent manner.

(a) HEK293T cells were treated with Wnt3a conditioned medium (CM), 70 μM xStAx-VHLL (xS-V) or 100 μM VH-289 as indicated for 24 h and then harvested for immunoblotting. (b) LoVo cells were treated with 50 μM xStAx (xS), 50 μM xStAx-VHLL (xS-V) or 100 μM VH-289 as indicated for 24 h and then harvested for immunoblotting. (c, d) LoVo (c) and SW480 (d) cells were treated with different amount of peptides as indicated for 24 h and then harvested for immunoblotting. The

relative β-catenin band intensity was quantified with ImageJ and normalized to GAPDH. xS: xStAx; xS-V: xStAx-VHLL. *p<0.05; **p<0.01.

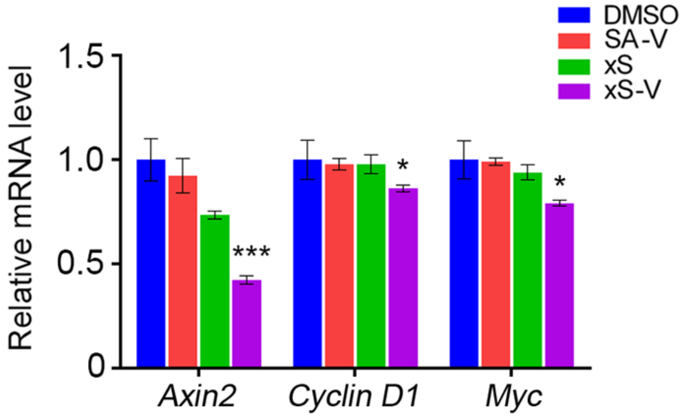

Supplementary Fig. S6. xStAx-VHLL suppresses the expression of Wnt target genes in LoVo cells-derived tumors.

The tumors derived from xStAx-VHLL-treated LoVo cells in nude mice were homogenized and extracted mRNA was subjected to qPCR analysis of the indicated Wnt target genes. Data from 3 independent experiments are displayed as the mean ± SD by one-way ANOVA. **p<0.01; ***p<0.001. SA-V: SAHPA1-VHLL; xS: xStAx; xS-V: xStAx-VHLL.

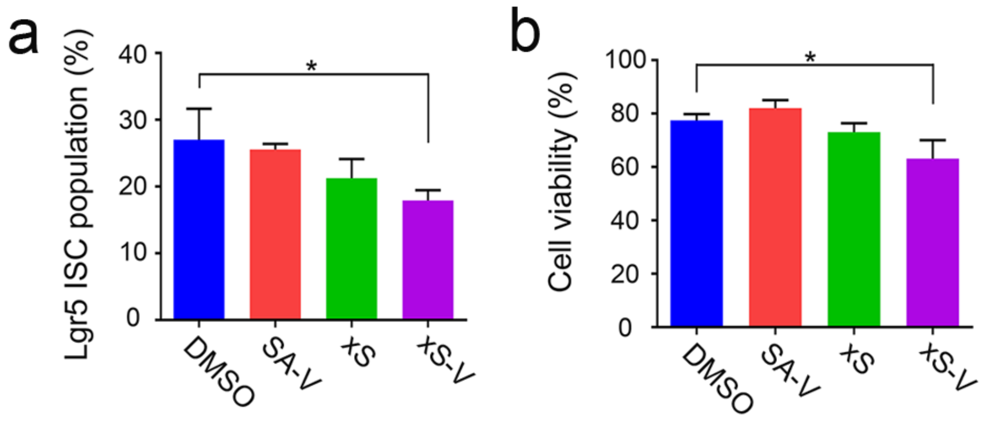

Supplementary Fig. S7. xStAx-VHLL restricts Lgr5^{+} intestinal stem cell population and cell viability in intestinal organoids.

The organoids derived from the small intestine of Lgr5-GFP mice were treated with 10 μM peptides for 36 h and then collected for CytoFLEX flow cytometry to count Lgr5^{+} intestinal stem cells (a) or viable cells (b). Data from 3 independent experiments are displayed as the mean ± SD by one-way ANOVA. *$p < 0.05$. SA-V: SAHPA1-VHLL; xS: xStAx; xS-V: xStAx-VHLL.

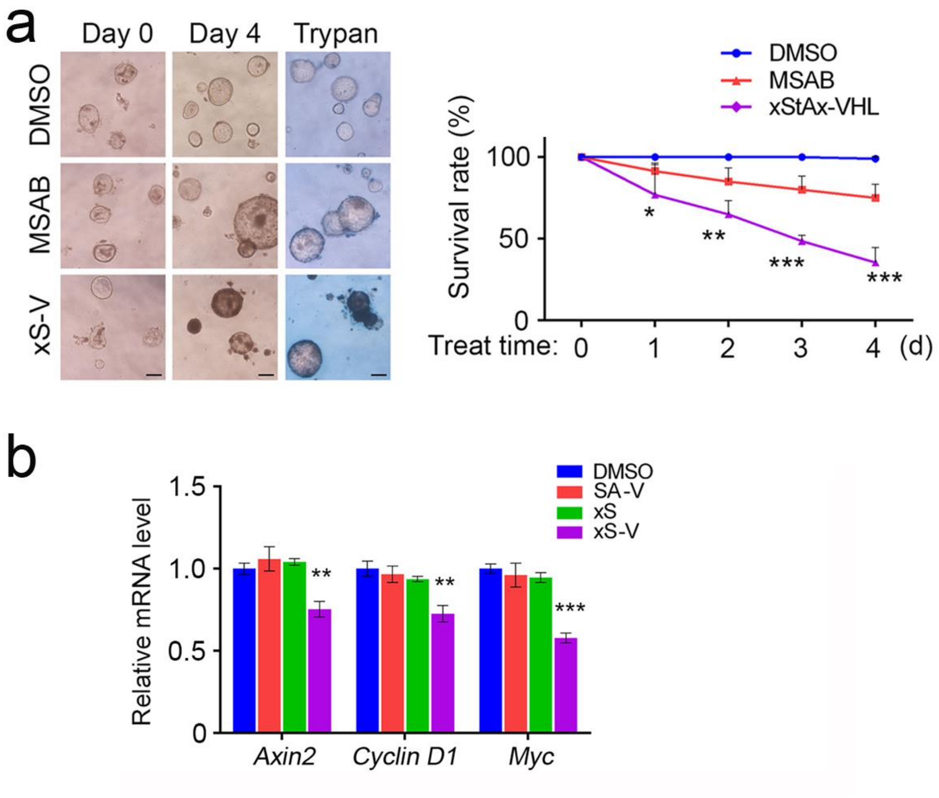

Supplementary Fig. S8. xStAx-VHLL inhibits the survival of mouse *APC*$^{-/-}$ organoids and Wnt target gene expression.

(a) Mouse *APC*$^{-/-}$ organoids were cultured with DMSO, 10 μM MSAB or 50 μM xStAx-VHLL for 4 days. Scale bar = 100 μm. Right panel: The survival rate of each group of *APC*$^{-/-}$ organoids was calculated. (b) *APC*$^{-/-}$ organoids were homogenized, and extracted mRNA was subjected to qPCR analysis of the indicated Wnt target genes. Data from 3 independent experiments are displayed as the mean ± SD by one-way ANOVA. *p<0.05; ***p<0.001. SA-V: SAHPA1-VHLL; xS: xStAx; xS-V: xStAx-VHLL.

a

Sample	Tumor Tissue Source	Age	Sex	Diagnosis	Original Tumor Occurrence	Stage	
						TNM	AJCC
C-01	Sigmoid colon	71	M	Adenocarcinoma	Sigmoid colon	T3N0M0	IIA
C-02	Rectum	37	F	Adenocarcinoma	Rectum	T4N1M0	IIB
C-03	Ascending colon	61	F	Adenocarcinoma	Cecum	T3N0M0	IIA
C-04	Sigmoid colon	62	M	Adenocarcinoma	Colon	T3N0M0	IIA
C-05	Sigmoid colon	57	M	Adenocarcinoma	Colon	T3N0M0	IIA
C-06	Rectum	44	M	Adenocarcinoma	Rectum	T4N1M0	IIB
C-07	Sigmoid colon	48	M	Adenocarcinoma	Sigmoid colon	T3N0M0	IIA
C-08	Rectum	41	M	Adenocarcinoma	Rectum	T1N0M0	I
C-09	Ascending colon	75	F	Adenocarcinoma	Colon	T3N0M0	IIA
C-10	Ascending colon	68	M	Adenocarcinoma	Colon	T3N0M0	IIA
C-11	Sigmoid colon	73	M	Adenocarcinoma	Colon	T3N1M0	IIIA
C-12	Sigmoid colon	68	M	Adenocarcinoma	Colon	T1N0M0	I

b

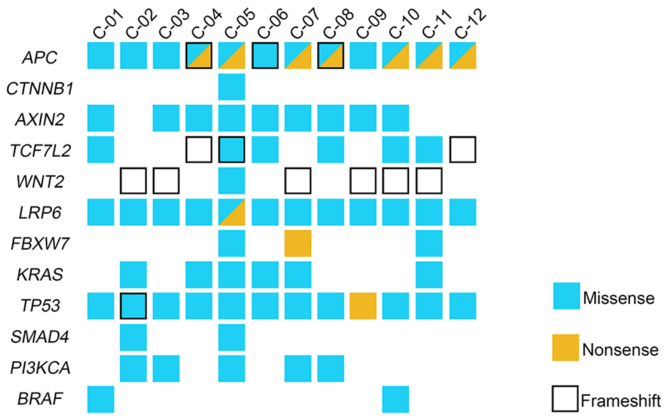

Supplementary Fig. S9. The CRC patient information and mutations of these tumor samples.

(a) The information of the 12 CRC patient samples was displayed.

(b) The genome DNA of tumor tissues from each CRC patient was subjected for whole exon sequencing. The missense mutation, nonsense mutation and frameshift mutation of the whole exon were analyzed.

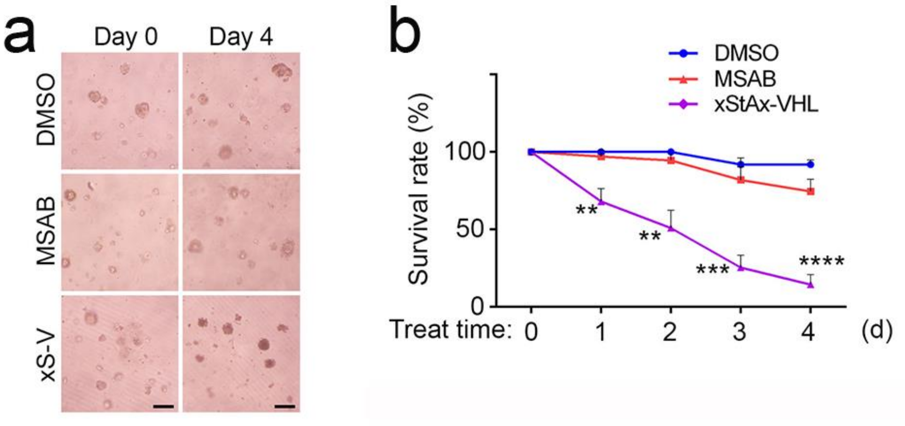

Supplementary Fig. S10. xStAx-VHLL inhibits the survival of human tumor organoids.

(a) The C-05 organoids were cultured with DMSO, 10 μM MSAB or 10 μM xStAx-VHLL for 4 days. Scale bar = 100 μm. (b) Right panel: The survival rate of each group of the organoids was calculated.
